# Tumour imaging by the detection of fibrin clots in tumour stroma using an anti-fibrin Fab fragment

**DOI:** 10.1038/srep23613

**Published:** 2016-03-24

**Authors:** Toshifumi Obonai, Hirobumi Fuchigami, Fumiaki Furuya, Naoyuki Kozuka, Masahiro Yasunaga, Yasuhiro Matsumura

**Affiliations:** 1 Division of Developmental Therapeutics, Exploratory Oncology Research & Clinical Trial Center, National Cancer Center, Kashiwa, Chiba, 277-8577 Japan; 2 Department of Integrated Biosciences, Graduate School of Frontier Sciences, The University of Tokyo, Kashiwa, Chiba, 277-8561 Japan

## Abstract

The diagnosis of early and aggressive types of cancer is important for providing effective cancer therapy. Cancer-induced fibrin clots exist only within lesions. Previously, we developed a monoclonal antibody (clone 102-10) that recognizes insoluble fibrin but not fibrinogen or soluble fibrin and confirmed that fibrin clots form continuously in various cancers. Here, we describe the development of a Fab fragment probe of clone 102-10 for tumour imaging. The distribution of 102-10 Fab was investigated in genetically engineered mice bearing pancreatic ductal adenocarcinoma (PDAC), and its effect on blood coagulation was examined. Immunohistochemical and *ex vivo* imaging revealed that 102-10 Fab was distributed selectively in fibrin clots in PDAC tumours 3 h after injection and that it disappeared from the body after 24 h. 102-10 Fab had no influence on blood coagulation or fibrinolysis. Tumour imaging using anti-fibrin Fab may provide a safe and effective method for the diagnosis of invasive cancers by detecting fibrin clots in tumour stroma.

It is widely recognized that the most effective way to reduce mortality rates in solid cancers is by early detection of the cancer. Therefore, diagnosing cancers at an operable stage (stage 2 or 3) is desirable. By contrast, some clinical cancers do not always need to be diagnosed; for example, senile patients with early stage prostate cancer are known to have no benefit from surgical or radiological treatment^1^. However, it is important to diagnose early stage or operable-stage cancers and truly aggressive malignant tumours for which treatment is critical.

Fibrin is the final product of the blood coagulation cascade^2^. Fibrin clots are not formed under normal conditions, but they accompany several pathological states, such as cardiac^3^ or cerebral^4^ infarction, injuries^5^, acute inflammation^6^, cancer invasion^7^ and metastasis^8^. Both intrinsic^9^ and extrinsic^10^ coagulation systems are known to be involved in tumour vascular permeability and tumour-induced blood coagulation, which result in the deposition of insoluble fibrin in various tumour tissues^10^,^11^,^12^,^13^,^14^. More erosive types of cancer exert greater destructive action^15^. If such cancer clusters erode adjacent normal or tumour vessels, haemorrhage may occur, followed by an immediate formation of fibrin clots *in situ* that stop the bleeding. These fibrin clots are subsequently replaced by collagen in a way that is similar to normal wound healing. Because of similarities between tumour stroma generation and wound healing, tumours have been referred to as “wounds that do not heal”^16^. Although there are many similarities between cancer-induced stroma and wound healing, the difference between the two is that the pathophysiological condition in cancer lasts for as long as cancer cells survive in the body. We have previously described the process of fibrin deposition in tumour stroma as the “malignant cycle of blood coagulation”^15^. We have also observed that fibrin deposition in glioma increases in a grade-dependent manner^11^. In addition, tissue factor (TF), which is the primary initiator of extrinsic blood coagulation, is now known to play important roles in tumour proliferation, invasion, and metastasis. TF is highly expressed on the surface of most human cancer cells^17^, and its expression is correlated with a poorer prognosis in various cancers^18^,^19^,^20^. Some non-malignant diseases form fibrin deposition, such as cardiac or brain infarctions and rheumatoid arthritis, but it is well established that in these diseases, fibrin clots form only at disease onset or during active states and disappear within a few weeks because of plasmin digestion and collagen replacement^11^. Fibrin deposition in non-malignant diseases is usually accompanied by symptoms that are related to the particular condition. By contrast, no symptoms are associated with tumour-related fibrin deposition. Therefore, the development of a method for the detection of fibrin clots is a reasonable effort from an oncological perspective.

In this context, we have developed an anti-fibrin antibody. We then developed a human/mouse chimeric antibody, 102-10 IgG, which can distinguish fibrin clots from fibrinogen, soluble fibrin, and D-dimer^11^. Although other anti-fibrin antibodies have been developed, none can react exclusively with fibrin clots, but they can react with fibrinogen, soluble fibrin, or D-dimer. Therefore, the production of a monoclonal antibody that can distinguish a fibrin clot from fibrinogen, soluble fibrin, and D-dimer would be a major breakthrough because all of these substances have common amino acid sequences. The specificity of 102-10 IgG differs from existing anti-fibrin antibodies (i.e., NYB-T2G1^21^,^22^ and MH-1^23^), and as a result of its unique properties, it is not neutralized by fibrinogen, soluble fibrin, or D-dimer in the bloodstream. The amino acid sequence of the epitope of 102-10 IgG is completely conserved in mammals, birds, amphibians, and fish (Basic Local Alignment Tool, BLAST). Therefore, 102-10 IgG against human fibrin clots can cross-react with mouse fibrin clots.

Numerous studies have reported tumour diagnoses using antibodies^24^,^25^. Because antibodies are able to bind specifically to their antigens, they have considerable potential as molecular imaging agents. For tumour imaging in particular, both tumour specificity and ease of use are strongly desired characteristics because the subjects are often outpatients. Although IgGs possess high specificity and avidity, IgG probes can take several days to provide acceptable imaging contrast because of their long blood circulation time. The plasma half-life of an IgG mainly depends on its size and biocompatibility^26^. In contrast, Fab fragment probes can extravasate more rapidly than their IgG forms and can reach the target organ and tissue within several hours of intravenous injection^26^,^27^. In addition, most infused Fab fragments are excreted from the body within a few days^24^.

In the present study, we developed a 102-10 Fab probe and evaluated its usefulness as a tumour imaging probe for medical use. In addition, we examined the influence of 102-10 Fab on blood coagulation and fibrinolysis in regard to adverse effects of the probe.

## Results

### Preparation and characterization of 102-10 Fab

**Figure 1:**
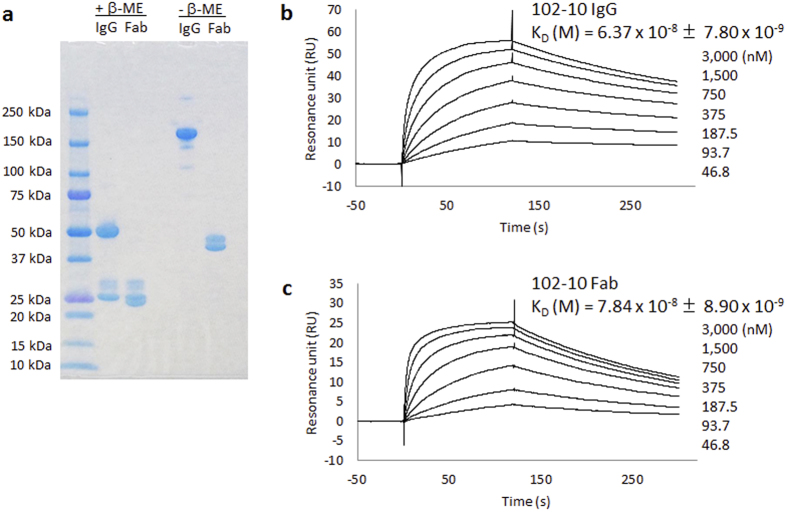
Characterization of 102-10 IgG and 102-10 Fab. (**a**) SDS-PAGE of 102-10 IgG and 102-10 Fab under reducing or non-reducing conditions. The binding activities of 102-10 IgG and Fab were evaluated by SPR analysis. Binding plots of 102-10 IgG (**b**) and Fab (**c**) by SPR imaging. Different concentrations of 102-10 IgG or Fab were injected onto a Biacore sensor chip containing an immobilized epitope peptide. An increase in the SPR signal was observed from 46.8 nM to 3,000 nM for both antibodies. The association of the Fab with the antigen was slower than that of the IgG. The dissociation of Fab from the antigen was more rapid than that of IgG. Each K_D_ value was represented as a mean value with standard deviation (n = 4); *P* < 0.05.

102-10 Fab was prepared from 102-10 IgG by papain digestion followed by affinity and size exclusion chromatography purification. SDS-PAGE under non-reducing conditions demonstrated that the molecular sizes of the original 102-10 IgG and the prepared 102-10 Fab were 150 kDa and 50 kDa, respectively (Fig. 1a). Under reducing conditions, the denatured antibody was separated into heavy and light chains. The heavy chains of 102-10 IgG and Fab had molecular weights of 50 kDa and 25 kDa, respectively, and both light chains had a molecular weight of 25 kDa. However, a few minor bands that originated from a glycosylated light chain were observed in both 102-10 IgG and Fab.

Surface plasmon resonance (SPR) analysis was conducted to compare the affinities of 102-10 IgG and Fab. However, the conventional SPR method is designed to evaluate the K_D_ values of antibodies with their corresponding soluble antigens. Therefore, SPR could not yield a K_D_ value for antibodies with insoluble antigens. Because the epitope sequence of 102-10 IgG has already been elucidated^11^, a GST-tagged epitope peptide was used as the ligand for SPR analysis. The resonance unit against the epitope with GST-tagged was subtracted that against GST-tagged alone in order to obtain the real K_D_ value against the epitope. The K_D_ values of 102-10 Fab and 102-10 IgG were 7.84 × 10^−8^ ± 8.90 × 10^−9^ M and 6.37 × 10^−8^ ± 7.80 × 10^−9^ M, respectively (Fig. 1b,c). Although affinity of 102-10 Fab decreased significantly compared to 102-10 IgG (*P* < 0.05), 102-10 Fab still retained its binding ability to the epitope peptide.

### Genetically engineered pancreatic cancer mouse model

**Figure 2:**
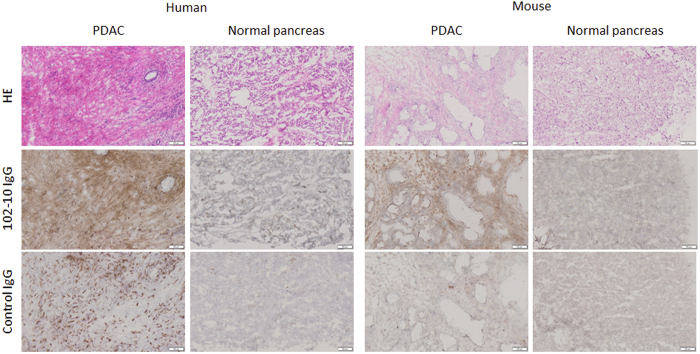
Histopathology and fibrin deposition in PDAC and normal pancreatic tissue. The HE staining are shown in upper panels, IHC using 102-10 IgG are shown in middle panels, and IHC using control IgG are shown in lower panels. Fibrin deposition was recognized only in human and mouse PDAC. Scale bar represented 100 μm.

Usually clinical human invasive cancer such as PDAC possesses abundant tumour stroma. However, only a small amount of cancer stromal tissue is formed in human cancer cell line-derived xenograft mouse models^28^. In this study, we focused on a genetically engineered PDAC mouse model that closely reflects the PDAC process in human^29^. We attempted to develop a LSL-Kras^G12D/+^; LSL-Trp53^R172H/+^; Ptf1a-Cre (abbreviated as KrasP53Ptf1a in figure legends), triple-mutation mouse that was expected to develop spontaneous PDAC naturally. Kras and Trp53 are major drivers of PDAC development, and Ptf1a is expressed at an early stage of pancreas development^30^. The presence of abundant interstitial tissue and fibrin deposition were anticipated in the mouse tumour tissue. Almost all the LSL-Kras^G12D/+^; LSL-Trp53^R172H/+^; Ptf1a-Cre mice developed PDAC within 8 weeks of birth, and their tumours were composed of not only cancer cells but also abundant stromal tissue (Fig. 2). In addition, to confirm the formation of deposited fibrin in the human and mouse PDAC, immunohistochemistry (IHC) using 102-10 IgG, which can cross-react with human and mouse insoluble fibrin, was conducted. Rituximab, an anti-human CD20 antibody, is a human/mouse chimeric IgG1, as is 102-10 IgG. The IHC using anti-human CD20 IgG was carried out as isotype control experiment. A fibrous staining was clearly observed in human and mouse pancreatic cancer tissues by the IHC. On the other hand, neither 102-10 IgG nor control IgG (anti-human CD20) could not detect such fibrous formation in both normal pancreatic tissues, although the IHC by the anti-human CD20 IgG showed a dot formation in human PDAC tissues, which may be CD20 molecule as described previously^31^. The amount of fibrin in mouse PDAC was less than that of human PDAC. Therefore, avidin-biotinylated peroxidase complex method was applied to fibrin staining of mouse pancreas in order to see the clear fibrin deposition. All animal experiments, including those with normal mice, were conducted at 10–21 weeks after birth.

### Tumour accumulation and biodistribution of fluorescence-labelled 102-10 Fab

**Figure 3:**
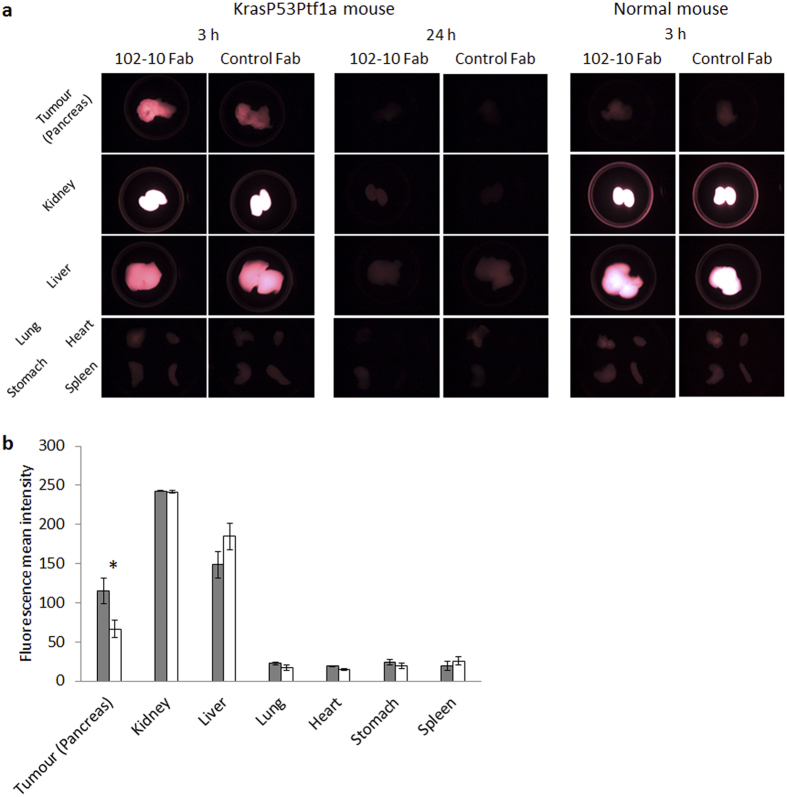
*Ex vivo* imaging of a KrasP53Ptf1a mouse bearing PDAC and a healthy normal mouse. (**a**) *Ex vivo* fluorescence imaging of each organ of a KrasP53Ptf1a mouse resected from a 102-10 Fab-AF or control Fab-AF 750-infused mouse 3 h (left two columns) and 24 h (middle two columns) after intravenous injection. Images of a healthy mouse 3 h after intravenous injection are shown in the right two columns. (**b**) Quantified fluorescence intensity of the tumour and each organ 3 h after intravenous injection of 102-10 Fab-AF 750 (grey bar) and control Fab-AF 750 (white bar). Each fluorescence intensity is represented as the mean value with standard deviation; *P < 0.05.

A near-infrared fluorophore, Alexa Fluor 750 (AF 750), was used to label 102-10 Fab, and 102-10 Fab-AF 750 was injected into LSL-Kras^G12D/+^; LSL-Trp53^R172H/+^; Ptf1a-Cre or normal mice via the tail vein. The tumour accumulation and biodistribution of 102-10 Fab-AF 750 were observed by *ex vivo* fluorescence imaging. Prepared rituximab-Fab can be used as the isotype control for 102-10 Fab and is hereafter referred to as the control Fab. Each Fab probe, which contained equivalent fluorophore molecules, was injected into LSL-Kras^G12D/+^; LSL-Trp53^R172H/+^; Ptf1a-Cre or healthy normal mice via the tail vein. *Ex vivo* fluorescence imaging was performed 3 or 24 h after intravenous injection (Fig. 3a). Compared with control Fab-AF 750, 102-10 Fab-AF 750 accumulated significantly in the PDAC. Because the kidney and liver are involved in the excretion of the probes^32^, a high level of fluorescence intensity was detected in those organs 3 h after intravenous injection for both 102-10 and control Fab-AF 750. However, neither 102-10 Fab-AF 750 nor control Fab-AF 750 accumulated in normal pancreases 3 h after intravenous injection or in other major organs, such as the lung, heart, stomach and spleen. The fluorescence intensity of both injected Fab probes was almost completely eliminated from the body 24 h after injection.

In fluorescence *ex vivo* imaging, the contour of an entire organ that was resected from the body was defined as the region of interest (ROI). This ROI was then analysed using imaging software to quantitatively compare the fluorescence intensity of 102-10 Fab-AF 750 with that of control Fab-AF 750 in each organ 3 h after intravenous injection (n = 3) (Fig. 3b). Because of the difficulty in distinguishing the PDAC area from the normal pancreas with the naked eye or by palpation, the entire pancreas was defined as an ROI of the tumour tissue. The calculated mean fluorescence intensities of 102-10 Fab-AF and control Fab-AF 750 were 115.2 ± 16.8 and 66.6 ± 11.5, respectively (*P* < 0.05). There were no significant differences in intensity between the fluorescent probes in other organs.

### Overlap of injected fluorescent 102-10 Fab and deposited fibrin

**Figure 4:**
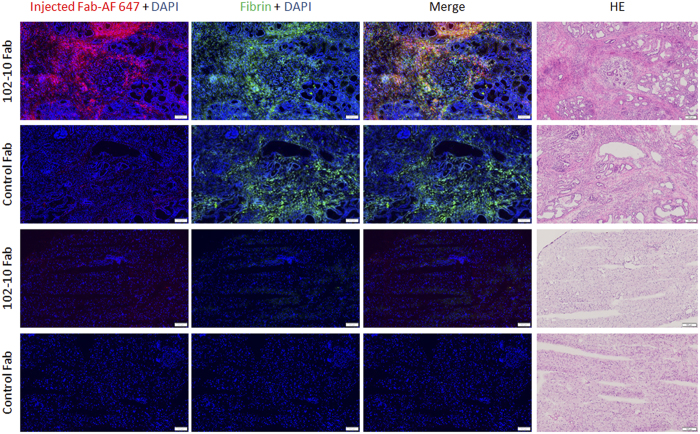
Localization of injected Fab probe and fibrin deposition in the PDAC or normal pancreas. The mouse PDAC (upper two rows) or normal pancreas (lower two rows) sections which were prepared from the mouse injected with 102-10 Fab- or control Fab-AF 647 probe were examined using fluorescence and optical microscopy. Red, blue and green indicate Fab probe, DAPI and fibrin deposition, respectively. Yellow indicates the overlap of the injected 102-10 Fab-AF 647 and deposited fibrin. The scale bar represents 100 μm.

To confirm whether the injected fluorescent 102-10 Fab reached the deposited fibrin clots in the PDAC tissue, we examined the intratumoural distribution of the fluorescent 102-10 Fab and control Fab. Frozen tissue sections were prepared from the pancreases of LSL-Kras^G12D/+^; LSL-Trp53^R172H/+^; Ptf1a-Cre or normal mice 3 h after intravenous injections of 102-10 Fab-AF 647 or control Fab-AF 647. 102-10 Fab-AF 647 was observed mainly in the fibrin-positive tumour stroma (Fig. 4). However, there was also a fibrin-positive tumour stroma area in the control Fab-injected tissue, while only a small amount of control Fab-AF647 was observed in that area. No fluorescence was observed in normal pancreatic tissue when 102-10 Fab-AF 647 or control Fab-AF 647 was administrated in normal mouse. Also IHC showed no fibrin deposition in normal pancreatic tissue. These results suggest that 102-10 Fab specifically binds to fibrin clots in the tumour stroma *in vivo*.

### Influence of 102-10 Fab on coagulation and fibrinolysis

A fibrin clot consists mainly of polymerized fibrin, which is produced by the limited digestion of fibrinogen by thrombin, followed by polymerization of the fibrin monomer^33^, and it is the final product of the blood coagulation system^2^. A fibrin clot is degraded by plasmin in the fibrinolysis system^2^. To verify the influence of 102-10 Fab on blood coagulation and fibrinolysis, fibrin gel formation and destruction assays were performed.

**Figure 5:**
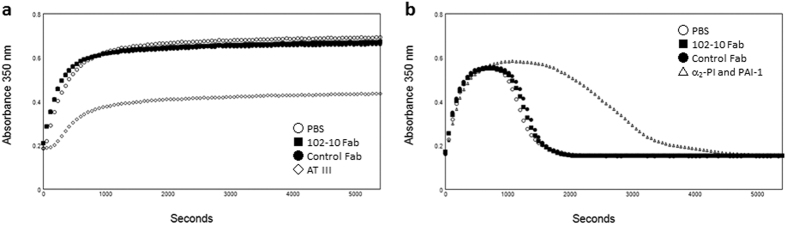
Influence of 102-10 Fab on coagulation and the fibrinolysis system. (**a**) Fibrin gel formation was initiated by mixing fibrinogen, thrombin, CaCl_2_, and Tween 80 with Fab fragments or coagulation inhibitor AT III according to a previously reported method^34^ with minor modifications. Turbidity was monitored by measuring the absorbance at 350 nm. (**b**) Fibrin gel degradation was monitored in a manner similar to that for fibrin gel formation by mixing fibrinogen, thrombin, CaCl_2_, Tween 80, PLG, and tPA with Fab fragments or fibrinolysis inhibitors (α_2_-PI and PAI-1).

In the fibrin gel formation assay, gel formation was monitored by measuring turbidity (Fig. 5a). Antithrombin III (AT III), which binds to thrombin and inhibits its enzymatic activity, was used as a negative control reagent. AT III significantly inhibited fibrin gel formation. By contrast, 102-10 Fab barely affected gel formation compared to the PBS control.

In the fibrinolysis system, a formed fibrin clot is degraded by plasmin, which is produced by the limited digestion of plasminogen (PLG) by a tissue-type plasminogen activator (tPA). In the fibrin gel degradation assay, PLG and tPA were added to the same clotting mixture as in the fibrin gel formation assay at the start of clot formation to simulate innate fibrinolysis^34^,^35^,^36^,^37^ (Fig. 5b). As a negative control, an α_2_-plasminogen inhibitor (α_2_-PI), which inactivates plasmin activity immediately by binding to the lysine binding site of plasmin, and a plasminogen activator inhibitor-1 (PAI-1), which inactivates tPA activity by binding to it, were added^38^. The addition of α_2_-PI and PAI-1 to the reaction mixture delayed lysis, and 102-10 Fab had no effect on fibrin gel degradation. In addition, control Fab did not exhibit delayed lysis. The binding of 102-10 Fab to fibrin affected neither blood coagulation nor fibrinolysis.

**Figure 6:**
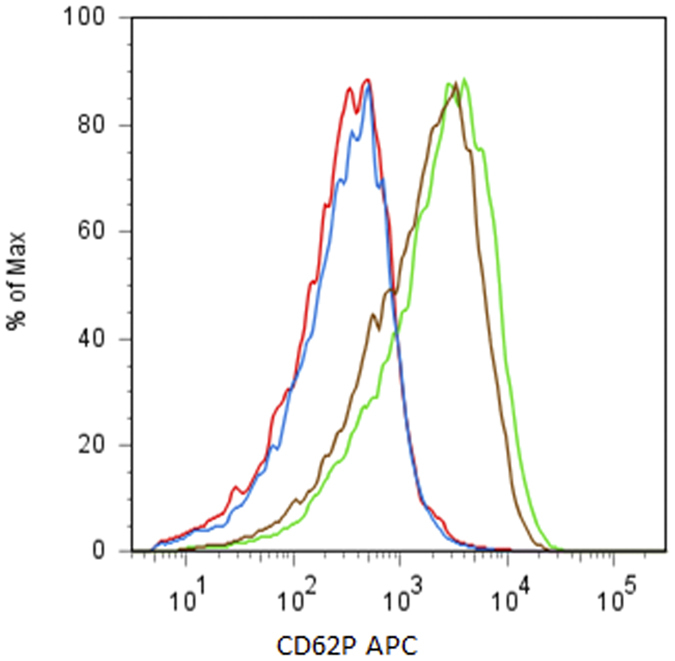
Platelet activation in 102-10 Fab- or control Fab-injected mouse blood. Activation of platelets gated with an anti-CD41 FITC positive fraction was observed by anti-CD62P APC reactivity using flow cytometry. The platelets prepared from 102-10 Fab- (red) and control Fab- (blue) injected mice expressed CD62P at a low level. Each platelet preparation was activated by the addition of thrombin specifically, 102-10 Fab (green) and control Fab (brown).

Although several anti-fibrin antibodies have been developed, some of them delay or inhibit fibrin gel formation in the presence of an anti-fibrin antibody, and these have been reported in previous studies^39^,^40^. Because the disruption of fibrin gel formation can lead to serious adverse effects, we examined the influence of 102-10 Fab on blood coagulation. First, we investigated the effect of 102-10 Fab on platelet activation. Platelet activation was measured by flow cytometry using CD41, which is a transmembrane glycoprotein that is expressed on platelets, and with CD62P, which is a platelet activation-dependent granule membrane protein and is also known as P-selectin. As shown in Fig. 6, the analysis of CD41-positive platelets prepared from mice injected with 102-10 Fab indicated that the expression level of CD62P was the same as that from platelets from mice injected with control Fab. However, platelets from both sources were activated by the addition of thrombin; therefore, platelets maintained a resting state until they were exposed to thrombin. Next, blood coagulation-related values were investigated. There were no significant differences regarding platelet count, activated partial thromboplastin time (APTT), prothrombin time (PT), and fibrinogen level between the 102-10 Fab and control Fab groups (Table 1). These results suggest that the injection of 102-10 Fab has no influence on blood coagulation *in vivo*.

## Discussion

In the present study, we examined the feasibility of using an anti-fibrin Fab fragment as a tumour imaging agent. Although the affinity of prepared anti-fibrin Fab, 102-10 Fab, was lower than that of original IgG, biding ability of 102-10 Fab against its epitope peptide was confirmed by SPR analysis. The feasibility of using 102-10 Fab as an imaging probe was evaluated using a LSL-Kras^G12D/+^; LSL-Trp53^R172H/+^; Ptf1a-Cre mouse model in which PDAC appears spontaneously. This spontaneous tumour exhibits remarkable fibrin deposition and cancer stromal-rich tissue that is similar to human PDAC. In the *in vivo* experiment, 102-10 Fab significantly accumulated in the tumour lesion 3 h after intravenous injection, and most of the 102-10 Fab probe was eliminated from the body within 24 h. These properties of selective targeting to the tumour tissue and rapid clearance from the body are desirable for an imaging agent, particularly if it is to be used for outpatients, for whom the tumour diagnosis testing should be completed within a few hours. In the distribution study, the infused, fluorescence-labelled 102-10 Fab appeared to accumulate selectively in tumour stroma where pronounced fibrin deposition occurred. The overlap of 102-10 Fab and deposited fibrin in the lesion strongly suggests that tumour accumulation of 102-10 Fab was due to specific binding to fibrin deposits in the tumour stroma.

Furthermore, for diagnostic use in clinical practice, safety issues should be considered because the diagnostic test would be applied to both healthy people and outpatients. IgG contains a constant Fc region, which triggers the immune response to a given antigen and may cause serious allergic reactions. Therefore, it is clear that a Fab lacking an Fc would be safer than an IgG in this regard. In addition, if a Fab conjugated with a radioisotope is used as a tumour imaging probe, the internal exposure levels to radionuclides would be reduced due to rapid renal excretion. In addition, if antibodies that bind blood coagulation-related factors are used clinically, concerns arise regarding bleeding or blood coagulation^41^,^42^,^43^. Therefore, an investigation of the effect on blood coagulation and fibrinolysis is necessary for this particular antibody. In the presence of 102-10 Fab, neither fibrin gel formation nor degradation was affected *in vitro*. Moreover, 102-10 Fab had no effect on blood coagulation *in vivo*. These results strongly suggest that this anti-fibrin antibody has no significant effect on bleeding or hypercoagulability.

The epitope sequence of 102-10 IgG is highly conserved in mammals, and we have confirmed that our anti-fibrin antibody cross-reacts with mouse, rat, and human fibrin clots. Therefore, the results of the present study reproducible in humans. In our previous report, we demonstrated that the higher the grade of glioma progression, the more fibrin deposition there is in the tumour tissue^11^. Therefore, we also expect to achieve a qualitative diagnosis of cancer by tumour imaging with 102-10 Fab. Furthermore, because a stabilized insoluble fibrin clot is a major component of a thrombus, detecting and imaging fibrin clots in the body may be useful for diagnosing other thrombotic disorders, such as pulmonary embolism and stroke.

We plan to conduct additional *in vivo* imaging experiments using radioisotope-labelled 102-10 Fab to confirm its clinical usefulness. Although a slight decline in the affinity of the prepared 102-10 Fab was observed, 102-10 Fab possessed adequate binding affinity to fibrin clots *in vivo*. Our investigation indicated that fibrin deposition in a cancer stroma is a promising target for tumour imaging and that 102-10 Fab has great potential as an imaging agent for detecting cancer tissue *in vivo*. The results of this study strongly support the potential for a cancer stromal targeting (CAST) diagnosis^15^,^44^.

## Methods

### Preparation of the 102-10 Fab fragment

We have developed a human/mouse chimeric anti-fibrin antibody, 102-10 IgG, using antibody engineering technology as previously described^44^. To prepare the 102-10 Fab fragment, 102-10 IgG was incubated with immobilized papain, a component of the Fab preparation kit (Thermo Fisher Scientific, Waltham, MA, USA), according to the manufacturer’s instructions. The digested antibody was separated from the immobilized papain by centrifugation, and the Fab fragment was separated from the undigested IgG and Fc fragments using Protein A (Thermo Fisher Scientific). Finally, size exclusion chromatography was conducted using Superdex 75 pg (GE Healthcare, Fairfield, CT, USA) to yield the purified Fab fragment. Using the same procedure, an anti-CD20 Fab fragment was prepared from Rituximab (Zenyaku Kogyo, Tokyo, Japan) as an isotype control antibody.

### Surface plasmon resonance analysis

Experiments were performed using a Biacore T200 instrument, CM5 biosensor chips, and amine coupling according to the manufacturer’s instructions (GE Healthcare). Kinetic evaluations were performed using an immobilized GST-tagged peptide (CNIPVVSGKECEEIIR), which contains the 102-10 IgG epitope (~100 RU) or GST tag alone as a control (~100 RU) in 10 mM sodium acetate at pH 5.5. The anti-fibrin antibody, 102-10 IgG or 102-10 Fab (46.8–3,000 nM), was injected using multi-cycle kinetics. Injections were administered at 30 μl per minute at 25 °C. An HBS-N buffer at pH 7.4 (GE Healthcare) was used as a running buffer, and 10 mM glycine-HCl at pH 1.5 was used as a regeneration buffer. Binding analyses were performed using the 1:1 binding model of the Biacore T200 evaluation software, version 1.0. The resonance unit against the epitope with GST-tagged was subtracted that against GST-tagged alone in order to obtain the real K_D_ value against the epitope. Each K_D_ value was represented as the mean value with standard deviation (n = 4).

### Immunohistochemistry

A human PDAC tissue section (OriGene Technologies, Rockville, MD, USA) and a normal human pancreatic tissue section (BioChain, Newark, CA, USA) were purchased commercially. Resected pancreatic cancer tissues were embedded in an optimal-cutting-temperature (OCT) compound (Sakura Finetek Japan, Tokyo, Japan) and were frozen at −80 °C. Frozen section samples were fixed using chilled acetone (Wako Pure Chemical, Osaka, Japan). In the staining of human PDAC and normal pancreas sections, after blocking with 5% skimmed milk (Becton Dickinson, Franklin Lakes, NJ, USA) at room temperature for 30 min, the section was incubated with HRP-conjugated 102-10 IgG or isotype control IgG, anti-CD20 antibody for overnight at 4 °C. The staining of mouse PDAC and normal pancreas sections were conducted with VECTASTAIN Elite ABC KIT (Vector Laboratories, Burlingame, CA, USA) according to the manufacturer’s instructions. Endogenous biotin of pancreas was blocked by using Avidin/Biotin Blocking Kit (Vector Laboratories). All reactions were visualized using a diaminobenzidine staining reagent (Dako, Glostrup, Denmark), and the samples were counterstained with hematoxylin (Muto pure chemicals, Tokyo, Japan).

### *Ex vivo* fluorescence imaging

To prepare the fluorescence-labelled 102-10 Fab and isotype control Fab, each Fab was conjugated with AF750 dye using the SAIVI rapid antibody labelling kit, Alexa Fluor 750 (Thermo Fisher Scientific). Each AF 750-labelled 102-10 Fab or control Fab was injected into mice via the tail vein at a dose of 90 μg. After 3 or 24 h, the mice were sacrificed under deep anaesthesia, and 30 ml of isotonic sodium chloride solution (Otsuka Pharmaceutical Factory, Tokushima, Japan) were injected into the circulatory system via the heart for perfusion. The lung, heart, stomach, spleen, kidney, liver and pancreas were resected from the body, and these organs were observed under fluorescent light using the OV110 *in vivo* imaging system (Olympus, Tokyo, Japan). Fluorescence imaging data were analysed using ImageJ 1.47 v for the quantitative assay. After image analysis, the pancreatic tumours were quickly embedded in an OCT compound (Sakura Finetek Japan) for IHC staining of fibrin deposits.

### Intratumoural distribution of injected fluorescent 102-10 Fab

102-10 Fab- and control Fab-AF 647 were prepared using the AF 647 protein labelling kit (Thermo Fisher Scientific), and each probe was injected into the tail vein of LSL-Kras^G12D/+^; LSL-Trp53^R172H/+^; Ptf1a-Cre or normal mice at a dose of 270 μg. Three hours after injection, the pancreas was resected under deep anaesthesia after perfusion through the heart, was embedded in an OCT compound (Sakura Finetek Japan) and was frozen at −80 °C. Frozen tissue sections were fixed using chilled acetone (Wako Pure Chemical). After blocking with 5% skimmed milk (Becton Dickinson) at room temperature for 30 min, the section was incubated with AF 488-conjugated 102-10 IgG for 1 hour at room temperature or for overnight at 4 °C. AF 488 labelled antibody was prepared using the Alexa Fluor 488 protein labelling kit (Thermo Fisher Scientific). Nucleus was stained using DAPI (Roche, Basel, Switzerland). After the staining, fluorescence imaging was conducted with fluorescence microscopy system, VS120 (Olympus).

### Fibrin gel turbidity assay

The turbidity assay was performed in quadruplicate according to a minor modification of the method of Kim *et al*.^34^ at 37 °C in a 96-well plate (Corning, Corning, NY, USA) using a SpectraMax Paradigm (Molecular Devices, Sunnyvale, CA, USA) as a plate reader. Turbidity was monitored once per minute at a 350 nm wavelength and was calculated as the mean value (n = 4) in a volume of 100 μl HBS at pH 7.4.

For fibrin gel formation, 2.0 mg/ml fibrinogen (Sigma, St. Louis, MO, USA); 5 mM CaCl_2_ (Wako Pure Chemical); 0.01% Tween 80 (MP Biomedical, Santa Ana, CA, USA); and 0.5 NIH unit/ml thrombin (Sigma) were added to 10 μg/ml Fab fragments or 0.3 unit/ml antithrombin (SLS Behring K. K., King of Prussia, PA, USA) as AT III.

For lysis of the fibrin gel, 2.0 mg/ml fibrinogen (Sigma); 5 mM CaCl_2_ (Wako Pure Chemical); 0.01% Tween 80 (MP Biomedical); 0.5 NIH unit/ml thrombin (Sigma); 0.2 μM PLG (Enzyme Research Laboratories, South Bend, IN, USA); and 0.3 nM tPA (Technoclone, Vienna, Austria) were added to 10 μg/ml Fab fragments or to a mixture of 0.10 μM α_2_-PI (Hematologic Technologies, Essex Junction, VT, USA) and 2.0 ng/ml PAI-1 (Prospec, East Brunswick, NJ, USA) as a negative control.

### Platelet activation assay

Platelet preparation and activation assays were performed according to the methods of Goodall and Appleby^45^ with minor modifications. Mouse blood was collected under deep anaesthesia in a tube containing 20 unit/ml heparin (Mitsubishi Tanabe Pharma, Osaka, Japan). The whole blood was centrifuged for 5 min at 500 × *g* to prepare the platelet rich plasma (PRP). The PRP was centrifuged again for 8 min at 300 × *g*. After the addition of 0.5 μM prostacyclin (Cayman Chemical, Ann Arbor, MI, US), the PRP was centrifuged for 5 min at 1300 × *g*. The platelet preparation was incubated for 30 min at 37 °C with PBS containing 0.1% bovine serum albumin (Roche Diagnostics, Basel, Switzerland); 2 mM EDTA (Dojindo, Kumamoto, Japan); 0.02 unit/ml apyrase (New England BioLabs, Hertfordshire, UK); and 0.5 μM prostacyclin after washing with the same buffer. The prepared platelet containing fraction was analysed by flow cytometry using Aria II (Becton Dickinson) after staining with anti-CD41 FITC (eBioscience, San Diego, CA, US) and anti-CD62P APC (eBioscience). Platelet activation was evaluated by the reactivity of anti-CD62P APC against the platelet fraction, which was gated with forward/side scatter characteristics and anti-CD41 FITC positive fractions.

### Measurement of blood coagulation-related values

All the blood coagulation-related values were measured by LSI Medience Corporation (Tokyo, Japan). APTT, PT, and fibrinogen level were measured with HemosIL SynthASil (Instrumentation Laboratory, Bedford, MA, US); HemosIL RecombiPlasTin 2G (Instrumentation Laboratory); and HemosIL Fibrinogen-C (Instrumentation Laboratory), respectively. These three variables were measured using ACL ELITE PRO (Instrumentation Laboratory). Platelet counts were measured with cellpack (Sysmex, Hyogo, Japan) using XT-2000iV (Sysmex).

### Animal models

Conditional LSL-Trp53^R172H/+^ (National Cancer Institute, Frederick, MD, US); LSL-Kras^G12D/+^ (a gift from Y. Kawaguchi, C Wright and D. Tuveson); and Ptf1a-Cre^46^ (a gift from Y. Kawaguchi, C Wright and D. Tuveson) strains were interbred to obtain triple-mutant animals on a mixed 129R1/C57BL/6 background. These triple-mutant mice spontaneously developed PDAC 8 weeks after birth. Commercially obtained C57BL/6JJms (Japan SLC, Shizuoka, Japan) was used as a control normal mouse. The study was approved by the Committee for Animal Experimentation of the National Cancer Center, Tokyo, Japan. All animal procedures were performed in accordance with the Guidelines for the Care and Use of Experimental Animals established by the Committee. These guidelines meet the ethical standards required by law and also comply with the guidelines for the use of experimental animals in Japan.

### Statistical analyses

SPR analysis, fluorescence *ex vivo* imaging and blood coagulation-related data were presented as the mean value with standard deviation from at least three independent experiments (Figs 1b,c and 3b and Table 1). A two-sided Student’s t-test was used to compare the two groups.

## Additional Information

**How to cite this article**: Obonai, T. *et al*. Tumour imaging by the detection of fibrin clots in tumour stroma using an anti-fibrin Fab fragment. *Sci. Rep.* **6**, 23613; doi: 10.1038/srep23613 (2016).

## Figures and Tables

**Table 1:** Blood coagulation-related values for 102-10 Fab- and control Fab-injected mice.

	**102-10 Fab**	**Control Fab**
Platelet (×10^4^/ml)	102.1 ± 12.0	108.4 ± 16.1
APTT (sec)	30.6 ± 1.7	33.9 ± 3.1
PT (sec)	7.8 ± 0.1	7.9 ± 0.2
Fibrinogen (mg/dl)	180.7 ± 9.9	205.3 ± 64.7

Each value is indicated with standard deviation (n = 3).
